# The role of general cognitive skills in integrating visual and linguistic information during sentence comprehension: individual differences across the lifespan

**DOI:** 10.1038/s41598-024-68674-3

**Published:** 2024-08-01

**Authors:** Florian Hintz, Cesko C. Voeten, Dorottya Dobó, Krisztina Sára Lukics, Ágnes Lukács

**Affiliations:** 1 Deutscher Sprachatlas, Philipps University of Marburg, Pilgrimstein 16, 35032 Marburg, Germany; 2 Max Planck Institute for Psycholinguistics, Nijmegen, The Netherlands; 3 Department of Linguistics, University of Pennsylvania, Philadelphia, PA USA; 4 Department of Biology, University of Pennsylvania, Philadelphia, PA USA; 5 University of Amsterdam, Amsterdam, The Netherlands; 6 Fryske Akademy, Leeuwarden, The Netherlands; 7 Department of Cognitive Science, Faculty of Natural Sciences, Budapest University of Technology and Economics, Budapest, Hungary; 8 MTA-BME Momentum Language Acquisition Research Group, Eötvös Loránd Research Network (ELKH), Budapest, Hungary; 9 Center for Mind, Brain and Behavior, Philipps University Marburg & Justus Liebig University Giessen, Marburg & Giessen, Germany

**Keywords:** Individual differences; language-vision interactions, Processing speed, Working memory, Human behaviour, Cognitive ageing

## Abstract

Individuals exhibit massive variability in general cognitive skills that affect language processing. This variability is partly developmental. Here, we recruited a large sample of participants (N = 487), ranging from 9 to 90 years of age, and examined the involvement of nonverbal processing speed (assessed using visual and auditory reaction time tasks) and working memory (assessed using forward and backward Digit Span tasks) in a visual world task. Participants saw two objects on the screen and heard a sentence that referred to one of them. In half of the sentences, the target object could be predicted based on verb-selectional restrictions. We observed evidence for anticipatory processing on predictable compared to non-predictable trials. Visual and auditory processing speed had main effects on sentence comprehension *and* facilitated predictive processing, as evidenced by an interaction. We observed only weak evidence for the involvement of working memory in predictive sentence comprehension. Age had a nonlinear main effect (younger adults responded faster than children and older adults), but it did not differentially modulate predictive and non-predictive processing, nor did it modulate the involvement of processing speed and working memory. Our results contribute to delineating the cognitive skills that are involved in language-vision interactions.

## Introduction

Spoken language comprehension occurs in vastly different contexts. Whereas unimodal contexts, such as listening to someone talk over the phone, entail the processing of a single input stream, multimodal contexts, such as being in a face-to-face conversation or comprehending speech with reference to the immediate visual surrounding, require the coordination of different input streams. In such contexts, listeners must quickly integrate the information derived from processing the spoken input with information derived from visual processing (e.g., the interlocutor’s facial movements^1^ or properties of the objects in the visual world^2^). The notion that listeners often predict words that are likely to come up next has gained considerable support over the past 20 years^3,4^. Moreover, it has become clear that during language-vision interactions comprehenders exploit cues in the spoken and visual modality for generating predictions about upcoming words^5,6^. The present study is concerned with individual variability in this behaviour. Specifically, we examine the contributions of general cognitive skills to integrating visual and linguistic information during predictive sentence comprehension.

Predictive processing is assumed to be a general organizing principle for human cognition^7–9^ such that across different modalities (e.g., vision, audition) the brain continuously tries to anticipate what is coming next. Such behaviour benefits fast interactions with our environment and supports learning via feedback mechanisms that code discrepancies between predicted and encountered input, helping finetune the prediction of future events. Language comprehension involves processing of visual and auditory input. It is therefore perhaps not surprising that prediction has become a core feature of many models and frameworks^4,10–13^. Given the rapid pace of speech (2–3 words per second), anticipating linguistic information may contribute to ‘keeping up’ with the speaker and enabling fast turn-taking, which often occurs as fast as 200 ms^14^.

Studies investigating prediction during language-vision interactions have often used the visual world paradigm^15^. In this paradigm, participants are presented with displays featuring visual objects, typically two or four, and spoken language related to the visual input. In most studies, participants’ eye movements are recorded and analysed. In a seminal study, Altmann and Kamide^16^ observed that participants looked at the picture of a cake already before it was mentioned, when listening to the sentence “The boy will eat the cake”. These eye movements most likely reflect that the participants predicted ‘cake’ as coming up next based on verb-selectional restrictions (the other co-present objects in the scene were inedible: train, ball, car).

An important task for experimental research is to explore the limits of prediction and to capture variability between individuals in order to define the cognitive architecture and mechanisms underlying human information processing^17,18^. With regards to predictive language processing, several visual world studies examined the influence of linguistic and general cognitive person variables on language-mediated anticipatory eye movements. For example, in samples of university students, Hintz et al.^19^ and Rommers et al.^20^ observed that listeners with larger receptive vocabularies and better production fluency showed stronger evidence for prediction than individuals who scored lower on the respective tests. Similarly, Özkan et al.^21^ reported that measures of early language production abilities were positively associated with variance in eye-movement-based measures of prediction in children, aged between 4 and 8 years. In terms of general cognitive skills, Özkan and colleagues also found that children with better working memory abilities were more likely to engage in prediction than children with limited working memory. The latter finding aligns well with the results by Huettig and Janse^22^, who tested individuals aged between 32 and 77 years and observed facilitatory effects of working memory capacity and nonverbal processing speed: Larger working memory capacities and better nonverbal processing speed were associated with increased likelihood of anticipatory looks to upcoming targets.

Contributions of general cognitive skills, such as working memory (WM) and nonverbal processing speed (ProSpeed), to language comprehension are predicted by most contemporary accounts^18,23^. However, such general cognitive skills (e.g., WM, ProSpeed) become particularly relevant during language-vision interactions, where spoken language processing needs to be coordinated with visual processing.

Working memory, assumed to reflect the ability to hold a limited amount of information temporarily active^24–26^, may—according to Huettig et al.^27^—function as a nexus during language-vision interactions where linguistically-derived and visually-derived information are bound together and maintained active for shorter periods of time^28,29^. WM capacity is often assessed using the Digit Span test^30–32^, where participants are presented with sequences of spoken digits, which they are instructed to remember and subsequently reproduce in the order they were encountered (forward version) or in reverse order (backward version). Sequence length increases as participants respond correctly. While forward Digit Span gauges the storage of information in short-term memory, backward Digit Span additionally requires the manipulation of short-term memory contents.

ProSpeed has been linked to variability in many cognitive tasks^33,34^. It is conceived of as an indicator for the speed with which individuals process different types of stimulus input and carry out mental operations. A common way of assessing ProSpeed are reaction time tasks, where participants are presented with visual or auditory stimuli to which they need to respond as quickly as possible^35^. During language-vision interactions, ProSpeed is likely to play an important role since these contexts require rapid auditory and visual processing, as well as the integration of both information streams.

There is plenty of evidence suggesting that individuals exhibit variability in WM and ProSpeed across the lifespan^31,32,34,36–40^. The distribution in both skills across the lifespan is best captured by a U-shaped curve, where developing and aging individuals perform worse than younger and middle-aged adults. Although previous reports demonstrated that WM and ProSpeed affect predictive processing during language-vision interactions in both developing and ageing individuals^21,22^, an important question that has not been addressed is whether the predictive power of these skills changes as a function of age. In other words, do WM and ProSpeed contribute to explaining variability in predictive processing during language-vision interactions differently in developing and ageing individuals?

In the present study, we used an individual-differences approach to address these questions. We recruited a large number of healthy Hungarian participants, including children, younger and middle-aged adults, and ageing individuals. Given the vast variability in age (and associated cognitive abilities), tasks were needed that could be completed by all participants. Since Digit Span and reaction time tests have previously been used in these populations^31,32,34,36–40^, we included forward and backward Digit Span and visual and auditory reaction time tests to assess WM and ProSpeed, respectively. In terms of measuring predictive language comprehension, we adapted a task previously developed by Hintz et al.^41^, henceforth referred to as Predictive Processing in the Visual World (PPVW) task. In this task, participants were presented with displays of two objects, one on each side of the screen, and a spoken sentence referring to one of them (Hungarian translation of, “The man yesterday *verbed* the *patient*”). In half of the trials, the patient could be predicted based on verb-selectional restrictions. Participants were instructed to press the button associated with the picture they think will be referred to, as soon as they knew. Participants’ reaction times relative to the onset of the spoken targets served as dependent variable. Note that it takes approximately 430 ms to program and launch a button press for a speeded task involving a choice component^41^. Our underlying assumption was that reaction times occurring before or shortly after (< 430 ms) spoken target onset are a sensitive index of predictive sentence comprehension. That is, we assumed that reaction times (just as eye movements) are preceded by attentional shifts toward the target object when encountering spoken cues related to the co-present visual objects and that reaction times (and eye movements) are overt instantiations of covert cognitive processes (i.e., attentional shifts). We return to this issue in the discussion.

Although Hintz and colleagues had administered this test in younger adults, it seemed likely that children and older adults would also be able to complete it^42,43^. Moreover, this task could be implemented as an online experiment straightforwardly and thus facilitated the recruitment of large numbers of participants required for an individual-differences approach.

Based on the results by Hintz et al.^41^, we predicted responses before spoken target onset or shortly thereafter on predictable trials as participants could anticipate which of the objects would be referred to. For non-predictable trials, we expected responses substantially later, as the target objects could not be predicted before their onset^41^. The critical question was whether variability in the difference between predictable and non-predictable conditions was explained by the four individual-differences predictors (visual/auditory RT tests, forward/backward Digit Span) and whether the difference varied as a function of age.

We used a statistical technique, quantile generalized additive mixed-effects models, that is suited for experiments where the dependent variable does not follow a normal distribution and where the effects of predictor variables may be nonlinear in nature. Moreover, in addition to examining the predictors’ effects on a single summary statistic of the dependent variable, such as the median (a robust analogue to the mean for data with non-normal distributions), as is done in most RT analyses, we explored whether the predictors’ effects differ when considering different portions of the data. Specifically, we additionally examined the predictors’ effects on the 10% and the 90% quantiles of the RT distribution in the visual world task, which reflect fast responses (10% quantile) and slow responses (90% quantile), respectively.

## Methods

### Participants

Child and adolescent participants were recruited from schools with the help of teachers. The majority of younger adult participants were recruited through university courses where they received credit for their participation. Adult participants were recruited through convenience sampling. Elderly participants (> 65 years) were recruited from retirement homes. Only participants with no history of neurodevelopmental disorders or cognitive impairment were included in the study. In the case of elderly participants, either the test results for the mini mental state examination^44^ test were provided by the institutions or the montreal cognitive assessment Test^45^ was administered by an experimenter. Participants under 18 were tested with the informed consent of their parents. Participants between 14 and 18 also provided their own informed consent, as did all adult participants, in accordance with the principles set out in the Declaration of Helsinki. The study was approved by the United Ethical Review Committee for Research in Psychology (EPKEB-2018/87).

We collected data from 605 participants. During the pre-processing stages, 124 participants were excluded (see section pre-processing). Thus, data from 487 participants were included in the analysis. The mean age of these participants was 29 years (SD = 18, range = 9–90); 349 were female, 117 were male and 21 preferred not to say. Fifty-one participants were left-handed, 417 right-handed and 18 indicated not to have a dominant hand. At the time of testing, participants had received on average 13.74 years of schooling (including primary and secondary education, bachelor’s, master’s and PhD university programs; SD = 5.00, range = 2–42).

### General procedure

The tests were administered as part of a larger test battery examining the relationships between language skills and other cognitive functions. All tests were programmed in PsychoPy^46^; data were collected via the Pavlovia online platform (pavlovia.org). Auditory stimuli were presented over headphones. Children and adolescents completed the tests in small groups in a quiet classroom under the supervision of an experimenter. Younger and middle-aged adult participants completed the tests online from their homes. Elderly participants (> 65 years) were tested under the supervision of an experimenter. The order of trials in each test was the same for each participant. In terms of test order, we provided participants with a suggestion, but they were free to choose a different order.

### Test descriptions

*Predictive processing in the visual world test (PPVW).* We designed a Hungarian version of the Dutch equivalent of the predictive processing in the visual world test developed and piloted by Hintz et al.^41^; it was similar to the task used by Mani and Huettig^42^. Participants were presented with transitive spoken sentences describing simple events (Table 1, for examples of predictable and non-predictable items) and two pictures, arranged on left- and right-hand sides of the computer screen—one of which was the target. On non-predictable trials, both objects fitted the semantic restrictions of the verb (paint: snake, apple); on predictable trials, only one of the two objects fitted the restrictions of the verb (turn off: torch, apple). The test consisted of 47 items (23 non-predictable, 24 predictable, see Appendix, for all items). All sentences were spoken by a Hungarian female speaker and recorded in a quiet environment. The sound intensity of all sentence recordings was equalized. We used the same drawings as used by Hintz et al.^41^, which were coloured versions of the drawings provided by Snodgrass and Vanderwaart^47^.

**Table 1:** Stimulus examples for predictable and non-predictable items in the PPVW task.

*Predictable*
*A férfi tegnapelőtt bekapcsolta az elemlámpát*
The man the day before yesterday turn PAST3SgDef the torch-ACC
‘The man turned off the torch the day before yesterday’
Images: apple, torch
*Non-predictable*
*A férfi tegnapelőtt lefestett egy kígyót*
The man the day before yesterday painted PAST3SgDef a snake-ACC
‘The man painted a snake the day before yesterday’
Images: snake, glasses

A trial proceeded as follows, a black fixation cross appeared in the middle of the screen against a white background for 1000 ms. Target and distractor objects were presented on the left and on the right of the screen. The position (left versus right) of the target versus the distractor object was counterbalanced across trials. The appearance of the two objects was synchronized to the onset of the spoken sentence; the target word was the final word in the sentence. We inserted the adverb ‘yesterday’ between agent and verb to provide participants with additional time to inspect the displays and retrieve the object names. There were approximately 2.18 s (SD = 158 ms, range = 1.83–2.64 s) between sentence onset and target word onset. Participants were instructed to press the left (left-hand object) or right (right-hand object) arrow on the keyboard to select the object that would be referred to in the sentence as soon as they knew. Their response terminated the trial and the next trial started.

Participants completed two practice trials (one non-predictable and one predictable) before the test phase (45 sentences, 22 non-predictable and 23 predictable). Presentation order of predictable and non-predictable trials was randomized. Response time (RT) was the difference between the onset of the target name and participants’ button press.

*Visual and auditory reaction time tests.* To test visual and auditory processing speed, we used reaction time tasks that measured speed of processing in the visual and auditory modalities for simple stimuli. Visual and auditory reaction time tests were implemented as two tasks of the same test, presented in fixed order, starting with the visual task. In the visual task, an image of a red ball with white spots appeared in the middle of the screen as the target stimulus. In the acoustic task, a 440 Hz pure tone was presented. In the visual task, participants were instructed to look at a blank grey screen and monitor the appearance of the red ball in the middle of the screen, and to press the spacebar as fast as possible upon appearance. In the auditory task, participants were instructed to press the spacebar as soon as they heard the tone. Both tasks consisted of 32 trials. Inter-stimulus intervals varied randomly between 1223 and 4988 ms for the visual task, and between 1041 and 4883 ms for the auditory task. Participants’ scores were the mean RTs in both task modalities.

*Digit span tests.* We used the forward (forward DS) and backward (backward DS) Digit Span tasks^31,32^ to measure short-term memory and working memory capacity. Forward and backward DS were administered as two tasks of the same test, starting with forward DS. Recordings of digits (from 1 to 9), spoken by a Hungarian male speaker, were presented in sequence. The length of these recordings varied between 548 and 862 ms. Sound intensities of the recordings were equalized. In both tasks, participants were instructed to reproduce (by typing out the numbers on the keyboard) the presented digit sequences in the encountered order (forward DS task) or in the reverse order (backward DS task). Digits were presented with a stimulus onset asynchrony of 1000 ms. The sequence length varied between 2–10 digits, and sequences were presented in incremental order of length. Within each length, participants were presented with four sequences; testing terminated after three incorrect responses within the same length. Forward and backward DS were operationalized as the longest sequence length with at least two correct responses.

### Data analysis

*Pre-processing.* The dataset initially contained data from 605 participants. However, due to technical or human failures, some participants had no data for one of the four individual differences tasks. This concerned 97 participants, who were removed. Next, we excluded all participants (n = 5) with mean RTs larger than 1500 ms in either the visual or the auditory reaction time test. Since these simple responses can be carried out within 250 to 500 ms in healthy participants^41^, average RTs beyond 1500 ms reflect that participants did not carry out the task seriously.

For the PPVW, we removed all participants who had negative mean RTs in the non-predictable condition. Since the verb semantics in the non-predictable condition did not provide information on which object would be referred to, responses before target onset were not possible. Eight participants consistently responded before target onset in the non-predictable condition, reflecting guessing or non-serious behaviour. These participants were removed. Furthermore, we removed all incorrect responses, remaining negative RTs in the non-predictable condition, and RTs shorter than − 2000 ms and larger than + 3000 ms. Following these exclusion processes, we checked how many trials each participant retained and excluded all participants with fewer than 34 trials in total (i.e., 75% of all trials), which led to further exclusion of eight participants. The statistical analysis was based on data from 487 participants who contributed 20,960 data points in total. Given 47 possible data points per participant (amounting to 22,889 in total), 1,929 data points (8.43%) were removed during pre-processing.

**Figure 1:**
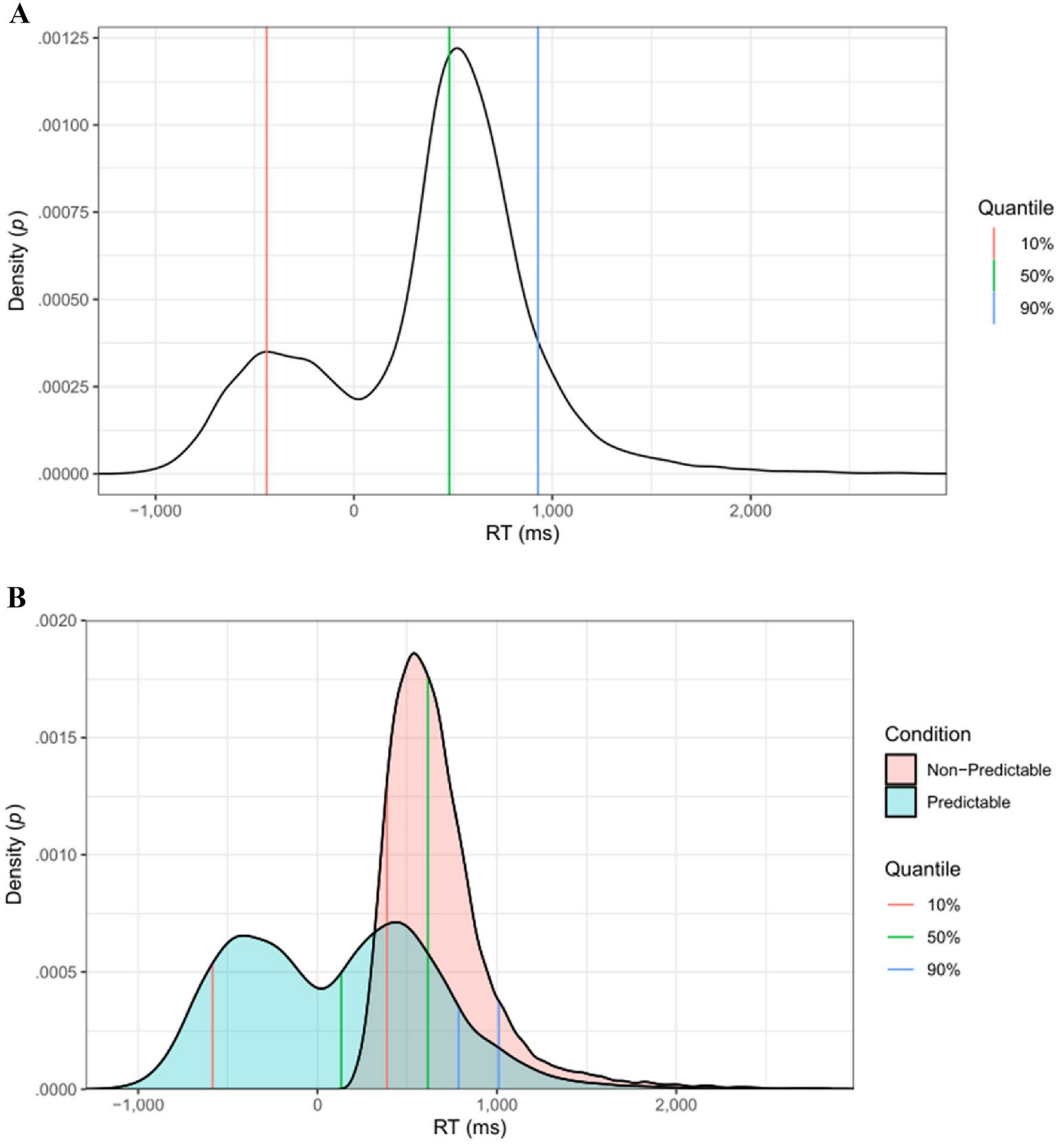
*Panel A*. Overall RT distribution in the PPVW task. Green (50%), red (10%) and blue (90%) vertical lines correspond to the quantiles targeted by our analyses. *Panel B*. RT distribution split out by condition (predictable vs. non-predictable).

*Statistical modelling.* Given the bimodal distribution of the RTs (Fig. 1), we analysed the data using quantile regression. Quantile regression is a distribution-free method of regression analysis. That is, instead of assuming a Gaussian dependent variable and fitting its mean value, quantile regression fits a specific quantile of a dependent variable, which is not assumed to follow a particular distribution. Fitting models for multiple quantiles enables us to examine the effects of our predictors (i.e., condition, age, visual/auditory RT, forward/backward DS) across the whole distribution of the data. That is, we can independently consider the median RT (the quantile regression analogue to a linear regression analysis), fast RTs (which represent the first mode in our data), and positive RTs (corresponding to the second mode). Our analytical approach is couched within the framework of *quantile generalized additive models*^48,49^, which enables the inclusion of random effects (e.g., participants) and penalized splines (in these data, age, which requires a more flexible predictor than a single straight line, and trial number) in the model.

We ran three quantile models. The first model targeted the 50% quantile of the data distribution, which corresponds to the median, which—if the data were normally distributed—corresponds to the overall mean as typically modelled in standard (regression) analyses. Furthermore, we ran a model targeting the 10% quantile, which corresponded closely to the first peak in our bimodal data distribution, and we ran a model targeting the 90% quantile of the data distribution, which corresponded closely to the second peak in our data (see Fig. 1, for an overview of the data distribution and the three quantiles). In all three cases, the models were fit to all data, in each case determining regression coefficients that produced the smallest residuals given the requested quantile.

Each model had the same dependent variable—RT—and the same predictors. These were fixed effects for Condition (predictable vs. non-predictable, sum-coded), forward DS (mean-centred), backward DS (mean-centred), visual RT (mean-centred), and auditory RT (mean-centred). In addition, interactions were included between Condition and the latter four individual-differences measures. Furthermore, we added a smooth term for age, defined as a thin-plate regression spline with maximally 15 basis functions. Within the generalized-additive-modelling framework, smoothing splines are used to fit predictors nonlinearly, with the amount of deviation from a straight line determined automatically from the data in a way that balances between overfitting and underfitting (here, based on the REML criterion). This allows the models to account for non-uniform effects of age in our dataset on the dependent variable. We additionally included terms for Condition, the four individual-differences measures, and the interactions between these, which we also allowed to vary nonlinearly with age using an approach called ‘varying-coefficient regression’^50^. The resulting nine difference smooths, relative to the main effect of Age, were given a point constraint passing through the origin, such that their intercepts would not compete with the parametric terms for the same nine predictors. In terms of random effects, we added random intercepts by participants, random slopes for Condition by participants. (while random effects are normally incompatible with quantile GAMs^49^, we were able to fit them as penalized parametric terms using the paraPen argument to gam, which is equivalent) and a smooth term for the trial number with appropriate penalties to the null space and the first derivative for this to be analogous in interpretation to a random factor. The latter was included to capture potential effects of fatigue, which may stack up in a nonlinear way as the experiment progresses.

The models as described above were fit to the three quantiles using function mqgam from R package qgam^51^. Estimation relied on the Extended Fellner-Schall optimizer, implemented by Wood and Fasiolo^52^. For the fixed effects in the models, significance of the results was assessed using Wald *z*-scores. For the smooth terms along age, we predicted each marginal smooth’s linear-predictor matrix onto a 100-point mesh, spanning the lowest to highest age values in our data. Multiplying these linear predictors by the model coefficients gave the fitted trajectory of the smooth. We assessed which parts of this trajectory were significant on the basis of 95% Bayesian credible intervals, computed using the approach by Wood^50^ (p. 293–294). We did not assess the random effects nor the smooth along trial numbers, since these nuisance terms were not of interest. We used function gam.check to confirm that the three models converged successfully and that all smooth terms had sufficient numbers of basis functions.

## Results

Table 2 lists the descriptive statistics for the PPVW task and the five predictor variables. In the PPVW, the average RT in the non-predictable condition was 675 ms. In the predictable condition, participants responded substantially earlier, on average 114 ms after target word onset. Given that it takes approximately 430 ms to program and launch a button press for a speeded task that involves a choice component^41^, these numbers suggest that participants prepared their response as soon as they recognized the unfolding target word in the non-predictable condition, but did so *before* spoken target onset in the predictable condition. The overall distribution of the RT data is visualised in Fig. 1, which suggests a bimodal distribution with a substantial portion of RTs occurring before target onset (i.e., in the predictable condition) and another peak after target onset (mostly RTs belonging to the non-predictable condition). The three vertical lines in Fig. 1 correspond to the three quantiles of the distribution targeted by our analyses. The green line corresponds to the 50% quantile (i.e., the overall median; analogous in interpretation to the mean known from traditional regression analysis). The red and blue lines correspond to the 10% and 90% quantiles of the distribution, respectively, reflecting fast and slow responses. The distribution plots in Fig. 1 and the standard deviations in Table 1 suggest substantial variability in the RTs, especially in the predictable condition.

**Table 2:** Descriptive statistics for dependent and independent variables for participants included in the analysis (n = 487).

Variable	M	SD	Range
PPVW (ms)			
*Predictable*	114	553	-1290 – 2990
*Non-predictable*	675	300	202 – 2929
Age (years)	29.25	17.78	8.70 – 89.60
Visual RT (ms)	351	132	231 – 1219
Auditory RT (ms)	334	83	197 – 801
Forward DS (score)	6.52	1.76	2 – 10
Backward DS (score)	5.30	1.97	2 – 10

**Figure 2:**
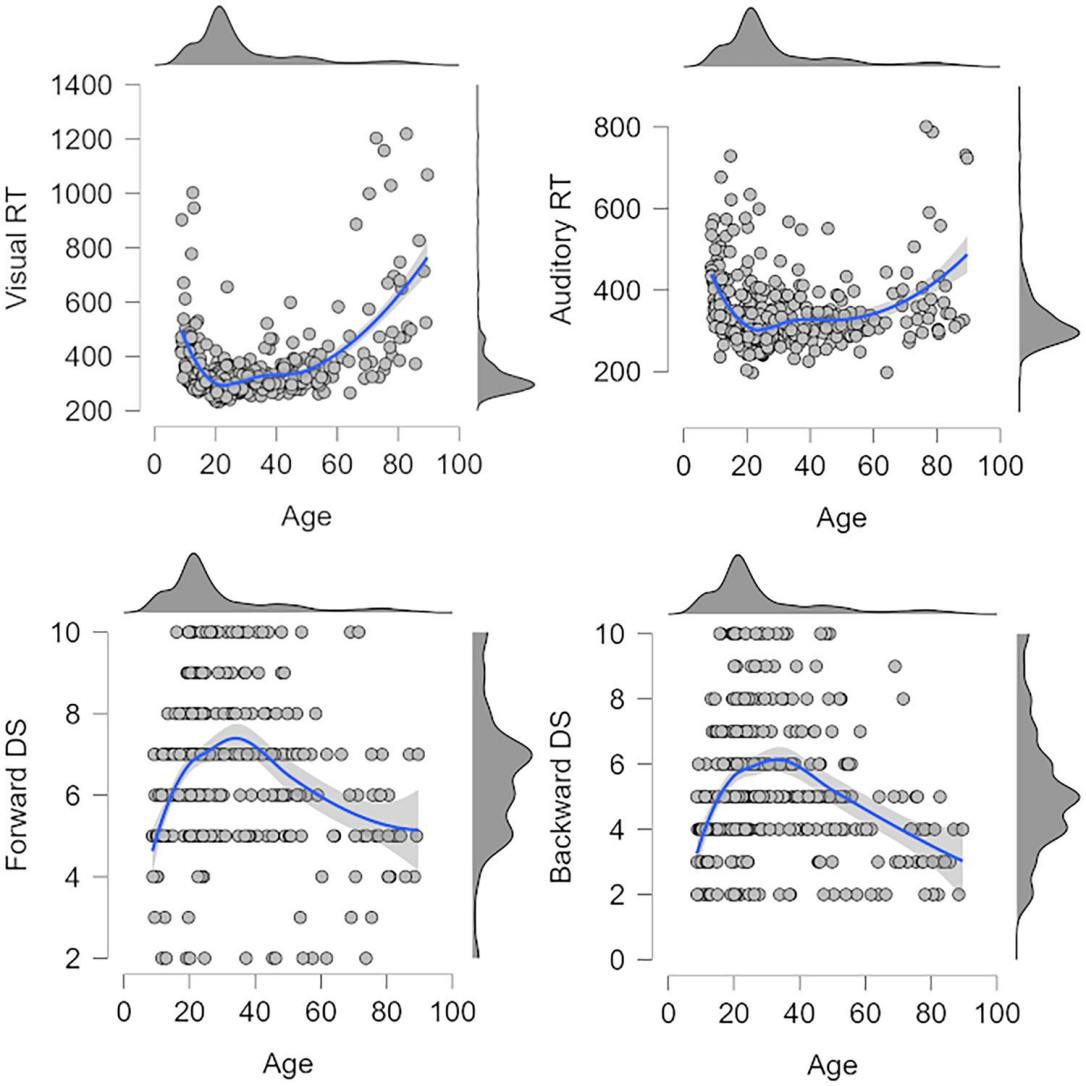
Scatter plots showing the relationships between age and the individual-differences predictors (visual/auditory reaction time, forward/backward Digit Span. Smoothed regression lines are accompanied by 95% credible intervals. Data distribution in each variable is visualized above and on the right of each scatter plot.

Mean RTs for the simple visual and auditory reaction time tasks were 351 ms and 334 ms. The average forward and backward DS were 6.52 and 5.30 digits. The descriptive statistics suggest massive variability between participants in all four tasks. The four panels in Fig. 2 visualize the relationships between age and the four individual-differences predictors by means of scatter plots. The fitted regression line in each plot suggests that, as expected, these relationships are best captured by U-shaped curves, illustrating that children and older adults performed poorer on the reaction time and working memory tests than younger adults did.

**Figure 3:**
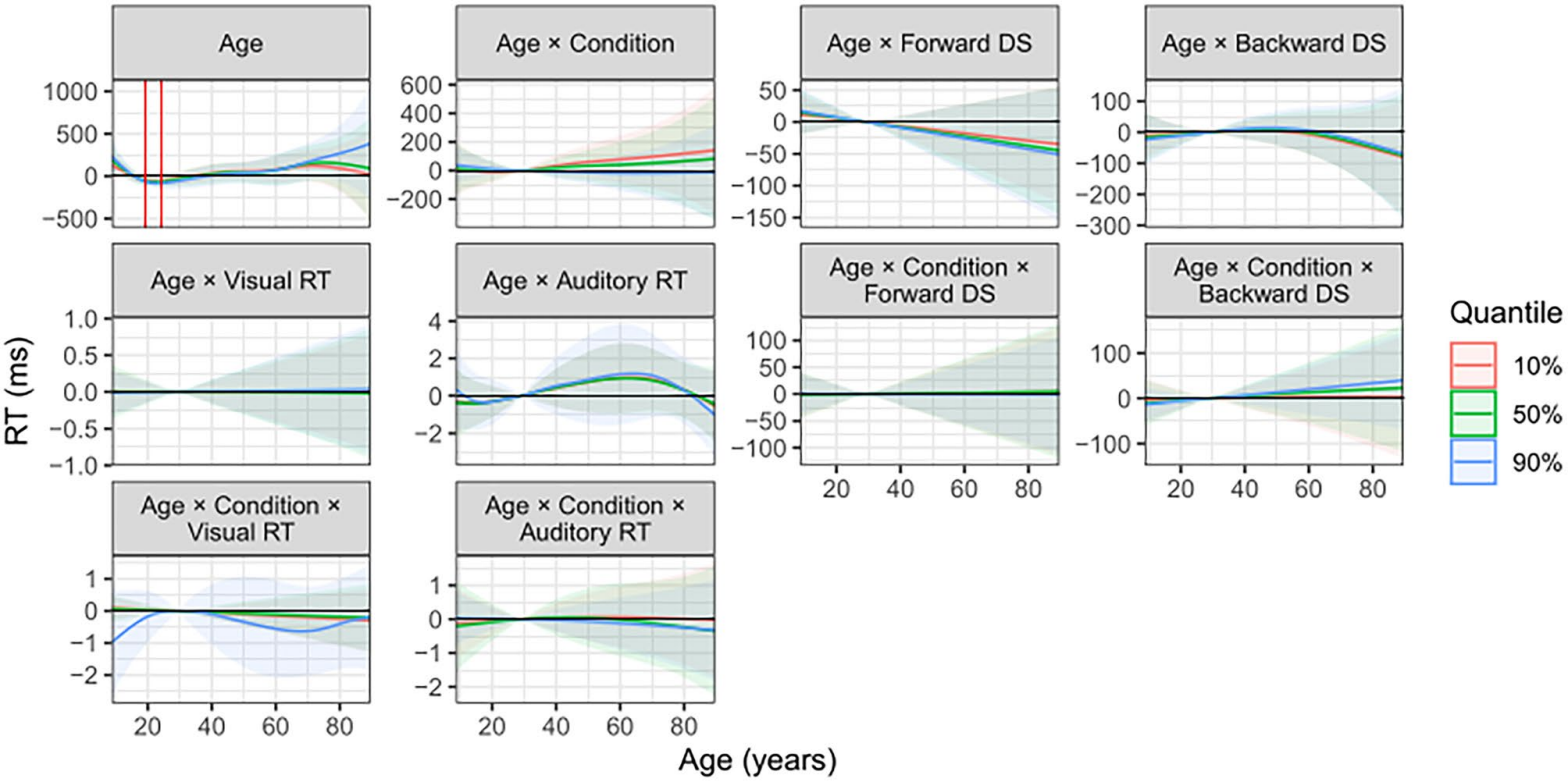
Results for the smooth terms in the quantile models.

Table 3 lists the results of the parametric terms for the quantile GAMs. These are interpretable in the same way as coefficients from a linear regression. The results indicate whether or not a parametric term significantly contributed to explaining variance in the dependent variable. Figure 3 plots the results for the smooth terms, i.e. Age and its interactions. These are marginal effects meaning they have the same interpretation as a regression coefficient, with the single difference being that the effect need not be (and indeed is not in our data) the same for all age values, but rather varies (smoothly) along the different age values. A visual heuristic for interpreting the plots in Fig. 3 is to focus on the trajectories of the three lines (each referring to one of the quantile models). Each line is surrounded by its 95% credible interval; if this credible interval does not include zero, that part of the trajectory represents a statistically significant effect. Below, we describe the results individually for each of the three quantile models and discuss their commonalities and differences in the Discussion.

**Table 3:** Results from the three quantile GAMs. Estimates are on the ms scale.

Predictor	Estimate (SE)	*z*	*p*
*Quantile* = *50% (distribution median)*			
Intercept	369.64 (12.13)	30.46	< .001***
Condition	-564.48 (40.22)	-14.04	< .001***
Forward DS	3.56 (7.19)	0.49	.62
Backward DS	-10.74 (8.46)	-1.27	.20
Visual RT	0.50 (0.13)	3.76	< .001***
Auditory RT	0.86 (0.26)	3.27	< .01**
Condition × Forward DS	5.07 (9.05)	0.56	.56
Condition × Backward DS	-15.25 (8.34)	-1.83	.07
Condition × Visual RT	0.40 (0.16)	2.57	.01*
Condition × Auditory RT	0.49 (0.23)	2.10	.04*
*Quantile* = *10% (fast responses)*			
Intercept	58.59 (11.07)	5.30	< .001***
Condition	-724.72 (39.26)	-18.46	< .001***
Forward DS	6.95 (6.54)	1.06	.29
Backward DS	-10.24 (7.70)	-1.33	.18
Visual RT	0.50 (0.12)	4.17	< .001***
Auditory RT	0.60 (0.25)	2.40	.02*
Condition × Forward DS	7.97 (8.64)	0.92	.36
Condition × Backward DS	-15.51 (7.93)	-1.96	.05
Condition × Visual RT	0.54 (0.15)	3.64	< .001***
Condition × Auditory RT	0.17 (0.21)	0.84	.40
*Quantile* = *90% (slow responses)*			
Intercept	757.53 (12.86)	58.92	< .001***
Condition	-358.17 (36.20)	-9.89	< .001***
Forward DS	0.36 (7.49)	0.05	.96
Backward DS	-11.21 (8.93)	-1.26	.21
Visual RT	0.47 (0.13)	3.46	< .001***
Auditory RT	0.96 (0.32)	2.95	< .01**
Condition × Forward DS	-2.68 (7.60)	-0.35	.72
Condition × Backward DS	-14.79 (6.77)	-2.19	.03*
Condition × Visual RT	0.62 (0.31)	2.00	.045*
Condition × Auditory RT	0.28 (0.17)	1.64	.10

### 50% quantile model

The 50% quantile, i.e. the median RT, represents the midpoint of the data distribution and is quantile regression’s closest analogue to the traditional linear regression. When the data deviate from normality, as ours do, the median provides a more robust estimate of the true overall effect.

We observed a strong effect of Condition: As suggested by the mean RTs, participants responded significantly faster in the predictable condition than they did in the non-predictable condition. In addition, we observed main effects of visual and auditory RTs on RTs in the PPVW task: The positive relationship suggests that participants with faster average responses on the visual and auditory RT tasks also responded faster on the PPVW task. Importantly, the effects of both individual-differences variables interacted with condition such that faster average responses on the visual and auditory RT tasks were associated with faster RTs in the predictable compared to the non-predictable condition. We observed no significant main effects for either forward or backward DS, nor were these found to stand in significant interactions with condition. While there was a trend for an interaction between condition and backward DS (better working memory skills associated with faster RTs in the predictable relative to non-predictable condition), there was no sign of an effect concerning forward DS.

The smooth terms (Fig. 3) show that, as expected, the nonlinear distribution of participants’ ages is represented well by a nonlinear spline. There was a significant main effect of Age on the overall RTs, highlighted in the left upper plot in Fig. 3 by means of the red vertical lines: in between these (i.e., participants aged 19 to 25 years), participants were significantly faster by 56 to 66 ms compared to the average participant. None of the other smooths involving age reached significance anywhere along it.

### 10% quantile model

For the 10% quantile, corresponding to the fastest RTs in the distributions, we observed a main effect of condition. The difference between predictable and non-predictable conditions was larger compared to the 50% quantile. The main effects of visual and auditory RT were similar to those in the 50% quantile model. Interestingly, while the effect of the visual RT interacted significantly with the effect of condition (as it did in the 50% quantile: faster average RTs were associated with faster RTs in the predictable relative to the non-predictable condition), this was not the case for auditory RT. Similarly, we observed no evidence for main effects of forward or backward DS, nor interactions of these with condition or an interaction between forward DS and condition. There was a strong trend for an interaction between condition and backward DS (better working memory skills associated with faster RTs in the predictable relative to the non-predictable condition).

The main smooth for age was again significant. Participants aged 20 to 24 years old responded significantly faster, by 51 to 55 ms, compared to the ‘average’ participant. As before, none of the other smooth terms, which included interactions of age with other predictors, showed a significant effect.

### 90% quantile model

As in the other two quantile models, we observed a main effect of condition for the 90% quantile, which was, however, smaller compared to the other quantiles. As before, we observed main effects of visual and auditory RTs and no main effects of forward and backward DS. As in the other models, visual RT significantly interacted with condition. In the 90% quantile model, we also observed evidence for a statistically robust interaction between condition and backward DS (better working memory skills associated with faster RTs in the predictable relative to the non-predictable condition).

Concerning the smooth of age, children aged up to 11½ years were slower by 231 to 141 ms, and ages 19 to 26 were faster by 74 to 78 ms than the ‘average’ participant. As in the other quantiles, no other smooth terms than the main effect were significant (Table 3).

## Discussion

Using an individual-differences approach, the present study tested the contribution of general cognitive skills to processing and coordinating visual and linguistic information during spoken sentence comprehension. A large sample of participants, varying substantially in age, completed tasks measuring visual and auditory processing speed as well as short-term and working memory. We observed that performance on these tasks co-varied with age, such that younger adults (from 18 up to their mid 20’s) performed better than children and older adults. We related participants’ performance on these tasks to their performance on a button-press-based version of the visual world paradigm measuring predictive and non-predictive sentence comprehension. We asked whether the explanatory power of visual and auditory processing speed and short-term and working memory for language-vision interactions changes across the lifespan. In doing so, we went beyond earlier studies^21,22^, which either tested developing or aging participants.

We observed robust evidence for condition differences across the three quantile models. That is, participants consistently responded earlier in the predictable than in the non-predictable condition when considering the 50%, the 10% and the 90% quantiles of the distribution. Although not the main focus, this finding demonstrates the converging validity of our adapted paradigm, which used reaction times instead eye movements as the dependent variable. In line with earlier research, our results thus show that developing and adult listeners readily integrate visual processing with spoken sentence comprehension to restrict the domain of subsequent reference^16,22,42^.

Our analyses revealed that this behavior is to a considerable extent subserved by ProSpeed—a result well in line with those reported by Huettig and Janse^22^ and predicted by contemporary accounts of language processing^18,23^. Unlike Huettig and Janse, who used principal component analysis to operationalize ProSpeed as a single variable based on multiple test scores, we submitted visual and auditory reaction time scores individually to our analyses. In all three models, main effects of visual and auditory reaction time emerged, suggesting that participants who responded faster on the ProSpeed tasks also responded faster on the sentence comprehension task. Crucially, we also observed that visual and auditory ProSpeed interacted with condition such that ProSpeed particularly benefitted processing in the predictable compared to the non-predictable condition. This finding is important since it highlights the specificity of the ProSpeed effects for predictive processing. Whereas visual reaction time showed this interaction across all three models, auditory reaction time showed this effect in the 50% but not in the 10% or 90% quantile models. Thus, predictive language processing in the visual world involves both visual and auditory ProSpeed, however, *visual* ProSpeed in particular plays a robust role, being observed not only for RTs that are typical in magnitude, but across the board. One may speculate that this is the case because visual processing typically precedes auditory processing in the scenarios tested in the visual world paradigm. As in most earlier studies, we presented the two objects before participants heard the cues (i.e., the verbs) relevant for generating predictions about the upcoming targets. It is thus plausible that rapid visual processing (e.g., retrieving semantic information about the depicted objects such as verb-selectional restrictions) is particularly beneficial for efficient mapping of vision-derived and language-derived information. Future research could follow up on this speculation by manipulating the time interval between display onset and onset of the relevant language cue to increase/decrease time pressure and/or by manipulating the visual set size (e.g., a larger number of objects may additionally increase the involvement of visual ProSpeed). Furthermore, future research could use a variety of visual and auditory ProSpeed tasks to further pinpoint the effects of ProSpeed on language-vision interactions.

Unlike in the study by Huettig and Janse^22^, we did not observe strong effects of WM. That is, our analyses did not reveal main effects of forward or backward DS, and the interaction between backward DS and Condition was only statistically reliable in the 90% quantile. One difference between the present study and that by Huettig and Janse is that our displays featured two instead of four objects. The demands on the WM system were therefore lower and although our participants differed massively in their WM capacities, these differences may not have affected performance on the PPVW task due to the overall lower demands. Interestingly, larger WM capacity was associated with faster responding on the PPVW task when considering the 90% quantile, featuring the slowest of all responses. That is, WM positively affected trials that may be considered momentary lapses of attention—participants with better compared to worse WM abilities were thus faster to recover WM contents for their response.

One may ask whether the difference in dependent variable in the PPVW task (reaction times vs. eye movements) and, relatedly, participants’ task contributed to the lack of WM effects. Indeed, the question to what extent active (e.g., instructions to click on one of the objects) and passive (no implicit viewing/response instruction) tasks affect (gaze) behavior in the visual world paradigm has been an ongoing debate^15,53–55^ since the paradigm’s re-invention^56^. The present study was not designed to contribute to solving this debate. We can therefore not exclude the possibility that our task (“Click on the object that will be referred”) and decision to record and analyze reaction times yielded different results compared to a version of the paradigm with eye movements as dependent variable and a passive look-and-listen task. Altmann and Kamide^16^ compared eye gaze across active (Experiment 1) and passive (Experiment 2) tasks and found—although the overall patterns were highly similar—that anticipatory eye movements emerged slightly earlier when participants carried out an active task. Against this background, it is conceivable that our participants were in general more ‘switched on’ as they were required to provide an overt response than the participants in Huettig and Janse^22^’s study who carried out a look-and-listen task.

In both settings, we may assume, though, that participants engage in previewing the visual scene during which they retrieve information about the two objects, which they keep in WM. They also engage in auditory processing and extract relevant cues from the spoken input. Thus, the choice to press the corresponding button *and* the (unconscious) decision to move the eyes to the target object are both preceded by a match between visually derived information held in working memory and information derived from the spoken input^27^. Whether in the present study anticipatory behavior emerged earlier than it would have when recording eye movements on a passive task is for future research to examine. However, concerning our focus on individual differences, it is unclear whether and how such a difference affected the engagement of general cognitive skills. Our hunch is that a setting that causes participants to be switched on (i.e., an active task) should increase rather than reduce the engagement of WM and ProSpeed. From this point of view, it appears unlikely that the lack of WM effects is due to the nature of the dependent variable and/or the active task. However, future research is required to test this conjecture.

The main focus of the present research was on the moderating effects of age. As expected, performance on the ProSpeed and WM tasks varied as a function of age in the form of a U-shaped curve: Young adults performed better than children, adolescents, middle-aged and older adults. Similarly, age also affected performance on the PPVW task, with younger adults (~ 19–26 years of age) generally responding faster compared to the mean of all participants (i.e., the ‘average participant’). However, age did not interact significantly with Condition nor did any other model term involving age show an effect. In other words, while it was the case that younger adults were fastest to carry out the PPVW task, this was a general advantage and did not differentially affect predictive and non-predictive sentence comprehension. Moreover, the predictive power of visual and auditory RT tasks and forward and backward DS did *not* change significantly across the lifespan and their relative contribution to explaining variance in predictive and non-predictive sentence comprehension was comparable across age groups. One may have predicted a different pattern. For example, adults, especially older adults, have more language experience, resulting in more entrenched lexical representations^57,58^ and stronger associative connections between lexical representations compared to developing language users. Moreover, there is ample evidence for the decline of older adults’ general cognitive skills (e.g., slower ProSpeed^34^, reduced WM capacity^32,36^). Against this background, one may have predicted that children rely more strongly on general cognitive skills than older adults to make up for the missing language experience. Older adults, on the other hand, may rely less strongly on general cognitive skills and more strongly on ‘linguistic heuristics’ (e.g., associative priming from predictive verbs to target objects may be more efficient in experienced than in developing language users). Instead, our results suggest that the extent to which ProSpeed and WM are involved in predictive sentence comprehension changes in step with age-related changes in these general cognitive skills.

At a more general level, our results provide support for previous experimental reports^22,59^ and contemporary accounts^23^ demonstrating the importance of ProSpeed for linguistic processing. As discussed above, we observed weaker effects of WM than Huettig and Janse^22^ and weaker effects than predicted by the WM model by Huettig et al.^27^. Given the nature of language-vision interactions and its proposed underlying architecture and mechanisms, it is likely that WM plays an important role in binding together visually derived and language-derived information and in keeping these information bundles active throughout the trial. For the methodological reasons discussed above (i.e., low load on the WM system), it would be premature to question WM’s involvement in language-vision interactions. Recall that our data showed strong trends for the critical interactions between WM and condition. An important methodological consideration for future research is to increase the number of objects from two to three or to increase sentence complexity/difficulty to increase the demands on the WM system, which may elicit (even) larger variability across participants.

To conclude, using an RT-based variant of the visual world paradigm and an individual-differences approach, we observed evidence for predictive sentence comprehension. Visual and auditory ProSpeed consistently contributed to explaining variance in this behaviour. The effects of WM in our data were restricted to a small portion of trials, subserving processing on ‘slow trials’. Participants’ ProSpeed and WM skills co-varied with age. Age had an overall effect on carrying out the PPVW task, but it did not modulate the extent to which ProSpeed and WM are involved in predictive sentence comprehension. These results contribute to mapping out the cognitive skills that affect visual and spoken language processing in language-vision interactions, and inspire further research on the role of cognitive skills and language experience in age-related changes in language processing.

### Supplementary Information

Supplementary Information.

## Data Availability

The raw data, analysis script, and item list are publicly available on OSF (https://osf.io/wy3bv/).
