# Supplementary material for: The role of general cognitive skills in integrating visual and linguistic information during sentence comprehension: individual differences across the lifespan
Source: Sci Rep. 2024 Aug 1;14:17797. doi: 10.1038/s41598-024-68674-3 (PMC11294566; doi:10.1038/s41598-024-68674-3)
Supplement: Supplementary file 1 — Supplementary Information. [file 41598_2024_68674_MOESM1_ESM.pdf]

Hungarian sentence	Condition	Verb	Target	Distractor	Translation
A férfi festett tegnapelőtt egy kígyót.	non-pred (p)	fest (paint)	kigyo (snake)	szemüveg (glasses)	The man painted a snake the day before yesterday.
A férfi látott tegnapelőtt egy harangot.	non-pred	lát (see)	harang (bell)	gyerek (child)	The man saw a bell the day before yesterday.
A férfi felborított tegnapelőtt egy biciklit.	non-pred	felborít (turn over)	bicikli (bicycle)	bőrönd (suitcase)	The man turned over a bicycle the day before yesterday.
A férfi kapott tegnapelőtt egy gyertyát.	non-pred	kap (receive)	gyertya (candle)	sál (scarf)	The man received a candle the day before yesterday.
A férfi ellopott tegnapelőtt egy órát.	non-pred	lop (steel)	ora (clock)	toll (pen)	The man stole a watch the day before yesterday.
A férfi rajzolt tegnapelőtt egy fúrót.	non-pred	rajzol (draw)	furo (drill)	bagoly (owl)	The man drew a drill the day before yesterday.
A férfi elrejtette tegnapelőtt a laptopot.	non-pred	elrejt (hide)	laptop (laptop)	lufi (balloon)	The man hid the laptop the day before yesterday.
A férfi eladott tegnapelőtt egy rúzst.	non-pred	elad (sell)	ruzs (lipstick)	ing (shirt)	The man sold a lipstick the day before yesterday.
A férfi rajzolt tegnapelőtt egy mágnest.	non-pred	rajzol (draw)	magnes (magnet)	jelzőlámpa (stoplight)	The man drew a magnet the day before yesterday.
A férfi kölcsönkért tegnapelőtt egy serpenyőt.	non-pred	kölcsönöz (borrow)	serpenyo (pan)	fejsze (ax)	The man borrowed a frying pan the day before yesterday.
A férfi vett tegnapelőtt egy pizzát.	non-pred	vesz (buy)	pizza (pizza)	tükör (mirror)	The man bought a pizza the day before yesterday.
A férfi kiválasztott tegnapelőtt egy hóembert	non-pred	kiválaszt (choose)	hoember (snowman)	sapka (cap)	The man chose a snowman the day before yesterday
A férfi vásárolt tegnapelőtt egy fogkefét	non-pred	vásárol (buy)	fogkefe (toothbrush)	kabát (jacket)	The man bought a toothbrush the day before yesterday
A férfi átadta tegnapelőtt a tollat.	non-pred	átad (hand over)	toll (feather)	kosár (basket)	The man handed over the pen the day before yesterday.
A férfi megkereste tegnapelőtt a fuvolát.	non-pred	keres (pay)	fuvola (flute)	könyv (book)	The man paid for the flute the day before yesterday.
A férfi lefilmezett tegnapelőtt egy hegedűt.	non-pred	filmez (film)	hegedu (violin)	szivar (cigar)	The man filmed a violin the day before yesterday.
A férfi letörölte tegnapelőtt a robotot.	non-pred	őriz (wipe)	robot (robot)	gitár (guitar)	The man wiped the robot the day before yesterday.
A férfi elpakolta tegnapelőtt a muffint.	non-pred	elpakol (put away)	muffin (muffin)	vállfa (hanger)	The man put away the cupcake the day before yesterday.
A férfi talált tegnapelőtt egy kulcscsomót.	non-pred	talál (find)	kulcscsomo (bunch of keys)	penztarca (packet of wallet)	The man found a set of keys the day before yesterday.
A férfi kifizette tegnapelőtt a dobot.	non-pred	fizet (pay)	dob (drum)	teniszütő (racquet)	The man paid the drum the day before yesterday.
A férfi megcsodált tegnapelőtt egy zongorát	non-pred	megcsodál (admire)	zongora (piano)	vitorlashajo (sailboat)	The man admired a piano the day before yesterday
A férfi megnézett tegnapelőtt egy gombát.	non-pred	megnéz (check)	gomba (mushroom)	sztetoszkóp (stethoscope)	The man checked a mushroom the day before yesterday.
A férfi lefotózott tegnapelőtt egy ruhát.	non-pred	fotóz (photograph)	ruha (dress)	kard (sword)	The man photographed a dress the day before yesterday.
A férfi bekapcsolta tegnapelőtt az elemlámpát.	pred (p)	bekapcsol (turn on)	elemlampa (flashlight)	alma (apple)	The man turned on the flashlight the day before yesterday.
A férfi vezetett tegnapelőtt egy autót.	pred	vezet (drive)	auto (car)	ablak (window)	The man drove a car the day before yesterday.
A férfi aláírta tegnapelőtt a szerződést.	pred	aláír (sign)	szerzodes (contract)	madár (bird)	The man signed a contract the day before yesterday.
A férfi megsimogatott tegnapelőtt egy kutyát.	pred	simogat (pet)	kutya (dog)	nap (sun)	The man petted a dog the day before yesterday.
A férfi fogott tegnapelőtt egy halat.	pred	fog (catch)	hal (fish)	sátor (tent)	The man caught a fish the day before yesterday.
A férfi elnyalt tegnapelőtt egy fagyit.	pred	nyal (lick)	fagyi (icecream)	vödör (bucket)	The man licked an ice cream the day before yesterday.
A férfi kifröcskölte tegnapelőtt a tejet.	pred	fröcsköl (spill)	tej (milk)	doboz (box)	The man spilled milk the day before yesterday.
A férfi elcserélt tegnapelőtt egy újságot.	pred	cserél (change)	ujsag (newspaper)	kerék (wheel)	The man changed a newspaper the day before yesterday.
A férfi kifacsart tegnapelőtt egy narancsot.	pred	facsar (squeeze)	narancs (orange)	öltöny (suit)	The man squeezed an orange the day before yesterday.

A férfi rágyújtott tegnapelőtt egy pipára	pred	rágyújt (smoke)	pipa (pipe)	busz (bus)	The man smoked a pipe the day before yesterday
A férfi letartóztatott tegnapelőtt egy tolvajt.	pred	letartóztat (arrest)	tolvaj (thief)	majom (monkey)	The man arrested a thief the day before yesterday.
A férfi ivott tegnapelőtt egy sört.	pred	iszik (drink)	sor (beer)	hátizsák (backpack)	The man drank a beer the day before yesterday.
A férfi megkent tegnapelőtt egy szendvicset.	pred	ken (eat)	szendvics (sandwich)	foci labda (ball)	The man ate a sandwich the day before yesterday.
A férfi kinyitotta tegnapelőtt az ajtót.	pred	kinyit (open)	ajto (door)	elefánt (elephant)	The man opened the door the day before yesterday.
A férfi felvett tegnapelőtt egy nadrágot.	pred	felvesz (put on)	nadrag (pants)	láb (foot)	The man put on a pair of trousers the day before yesterday.
A férfi megnyerte tegnapelőtt a kupát.	pred	nyer (win)	kupa (cup)	béka (frog)	The man won the cup the day before yesterday.
A férfi sütött tegnapelőtt egy tortát.	pred	süt (bake)	torta (cake)	hegy (mountain)	The man baked a cake the day before yesterday.
A férfi ültetett tegnapelőtt egy virágot.	pred	ültet (plant)	virag (flower)	bolygó (planet)	The man planted a flower the day before yesterday.
A férfi megterítette tegnapelőtt az asztalt.	pred	megterít (set)	asztal (table)	olló (scissors)	The man set the table the day before yesterday.
A férfi lekárpitozta tegnapelőtt a kanapét.	pred	kárpitoz (upholster)	kanape (couch)	hajó (ship)	The man upholstered the sofa the day before yesterday.
A férfi kicserélte tegnapelőtt az izzót.	pred	kicserél (change)	izzo (lightbulb)	ásó (shovel)	The man changed the light bulb the day before yesterday.
A férfi leborotválta tegnapelőtt a szakállát.	pred	borotvál (shave)	szakall (beard)	pulcsi (sweater)	The man shaved his beard the day before yesterday.
A férfi kitapétázta tegnapelőtt a falat.	pred	tapétáz (decorate)	fal (wall)	vonat (train)	The man decorate the wall the day before yesterday.
A férfi kitűzte tegnapelőtt a zászlót.	pred	kitűz (put up)	zaszlo (flag)	seb (wound)	The man put up the flag the day before yesterday.
